# The Effect of Blood Selenium Level on the pCR Rate in Breast Cancer Patient Receiving Neoadjuvant Chemotherapy

**DOI:** 10.3390/cancers17050839

**Published:** 2025-02-28

**Authors:** Marek Szwiec, Joanna Tomiczek-Szwiec, Wojciech Marciniak, Róża Derkacz, Tomasz Huzarski, Cezary Cybulski, Jacek Gronwald, Karolina Osowiecka, Robert Sibilski, Steven A. Narod, Jan Lubiński

**Affiliations:** 1 Department of Surgery and Oncology, University of ZielonaGóra, Zyty 28, 65-046 Zielona Góra, Poland; szwiec72@gmail.com (M.S.); r.sibilski@wlnz.uz.zgora.pl (R.S.); 2 Department of Biology and Genetics, Faculty of Medicine, University of Opole, Oleska 48, 45-052 Opole, Poland; tomiczek.onk@gmail.com; 3 Clinical Department of Oncological Gynecologyat the Oncology Centre in Opole, 45-061 Opole, Poland; 4 Read-Gene, Grzepnica, ul. Alabastrowa 8, 72-003 Dobra, Poland; wojciech.marciniak@read-gene.com (W.M.); roza.derkacz@read-gene.com (R.D.); 5 Department of Clinical Genetics and Pathology, University of ZielonaGóra, ul. Zyty 28, 65-046 Zielona Góra, Poland; tomasz.huzarski@pum.edu.pl; 6 Department of Genetics and Pathology, International Hereditary Cancer Center, Pomeranian Medical University in Szczecin, ul. UniiLubelskiej 1, 71-252 Szczecin, Poland; cezarycy@pum.edu.pl (C.C.); jacek.gronwald@pum.edu.pl (J.G.); 7 Department of Psychology and Sociology of Health and Public Health, School of Public Health, University of Warmia and Mazury in Olsztyn, Al. Warszawska 30, 11-041 Olsztyn, Poland; karolina.osowiecka@uwm.edu.pl; 8 Women’s College Research Institute, Toronto, ON M5S 1B2, Canada; steven.narod@wchospital.ca; 9 Dalla Lana School of Public Health, University of Toronto, Toronto, ON M5T 3M7, Canada

**Keywords:** selenium, neoadjuvant chemotherapy, breast cancer, pathological complete response

## Abstract

Purpose: Among patients treated with neoadjuvant chemotherapy (NAC), a high survival rate is observed for those who experience a pathological complete response (pCR). Various tumor factors are predictive of a pCR, but few host factors have been studied.We sought to inquire whether or not a patient’s blood selenium level prior to treatment was predictive of a pCR. Methods: We studied 329 women diagnosed with primary invasive breast cancer who were treated with neoadjuvant chemotherapy (NAC). We included patients with HER2-positive (*n* = 183) or triple-negative breast cancer (*n* = 146). Blood was collected before the initiation of treatment. Blood levels of selenium were quantified by mass spectroscopy. Each patient was assigned to one of three tertiles based on the distribution of blood selenium levels in the entire cohort. Patients with triple-negative breast cancer (TNBC) were treated with a range of combination chemotherapies. Patients with HER2-positive breast cancer received anti-HER2 treatment based on trastuzumab alone or trastuzumab and pertuzamab. After treatment, each patient was classified as having pCR or no pCR. Results: In the entire cohort, the pCR rate was 59.0% for women in the highest tertileof blood selenium (≥107.19 μg/L) compared to 39.0% for women in the lowest tertile (≤94.29 μg/L) (*p* = 0.003). Conclusions: A high selenium level is predictive of pCR in women treated for HER2-positive or triple-negative breast cancer. If confirmed, this observation may lead to a study investigating if selenium supplementation improves pCR rates and survival in breast cancer women receiving NAC.

## Simple Summary

This study aimed to examine the relationship between pCR rate and blood selenium level in a group of patients with HER2-positive breast cancer and TNBC who received neoadjuvant chemotherapy (NAC). Among the many clinical and histopathological factors assessed, the following had a statistically significant effect on the pCR rate: tumor size, Ki67 status, BMI and selenium level. In the entire cohort, the pCR rate was 59.0% for women in the highest tertile of blood selenium (≥107.19 μg/L) compared to 39.0% for women in the lowest tertile (≤94.29 μg/L) (*p* = 0.003). The association was much stronger for those with triple-negative cancer (OR = 6.70) (95% OR 2.18–20.56, *p* = 0.001) than for those with HER2-positive cancer (OR = 1.65) (95% OR 0.70–3.88, *p* = 0.25). To our knowledge, this is the first study to demonstrate an association between selenium levels in whole blood and pCR after neoadjuvant chemotherapy.

## 1. Introduction

Neoadjuvant chemotherapy (NAC) is now commonly used for the treatment of breast cancer and is the preferred treatment for HER2-positive or triple-negative breast cancer (TNBC). Pre-operative chemotherapy is comparable to adjuvant chemotherapy in terms of overall survival (OS) and disease-free survival (DFS) [1,2,3,4]. NAC is often given in locally advanced breast cancer to enable breast-conserving surgery [5,6]. When axillary lymph nodes are involved, NAC is often used to down-stage the axillary nodes and perform a sentinel lymph node procedure [7,8].

Another important indication of NAC is to assess the response to treatment, given that pCR predicts patient prognosis and survival [9,10,11]. In general, adjuvant therapy is offered post-surgery to patients who fail to achieve a pCR. In the case of HER2-positive breast cancer in the absence of pCR after NAC, post-operative treatment with TDM1 (trastuzumab emtansine) is recommended [12]. Similarly, in TNBC, the lack of pCR after NAC is an indication for capecitabine [13].

NAC is recommended for HER2-positive patients if the tumor size is 2 cm or greater or if the lymph nodes are positive [14]. For triple-negative breast cancers, NAC is recommended for tumors larger than 1 cm [14].

The frequency of achieving pCR is lower in ER-positive and HER2-negative breast cancers than in triple-negative or HER2-positive breast cancers [15,16,17]. Several other tumor factors predict the likelihood of achieving pCR [18,19]. Factors that have a negative impact include lobular histology [20,21] and expression of the estrogen receptor [19,22]. Factors that have apositive impact include a high Ki67 proliferation index [23,24,25] and high tumor grade [26,27]. In general, the response to NAC depends on the molecular subtype of breast cancer: the pCR rate for HER2-positive breast cancers approaches 60%; for TNBC ranges from 40 to 50%; and for luminal breast cancers is approximately 12% [6,28,29].

Other factors that may have a potential impact on achieving pCR are still being investigated, including germline mutations in the *BRCA1*, *PALB2* and *CHEK2* genes [30,31]. In the GeparSixto study, pCR rates with carboplatin were higher in *BRCA1* mutation carriers than in non-carriers [32]. A study in Polish patients showed a low pCR rate in patients with *CHEK2* mutations [33]. Contrasting results were obtained in a Chinese study, where in patients with the *CHEK2* founder mutation H371Y had a significantly higher pCR rate than non-carriers (33.3% versus 19.5%, *p* = 0.03) [34]. There are no data yet on the influence of germline mutations in the *PALB2* gene on the effectiveness of NAC. Other factors that have been reported to be associated with pCR include obesity, metabolic syndrome, dyslipidemia, the neutrophil-to-lymphocyte and the platelet-to-lymphocyte ratio [35,36,37,38].

We have previously reported an association between low serum selenium levels and poor survival in breast cancer patients [39,40]. Selenium isimportant in many metabolic pathways. It is a component of many selenoproteins. including the antioxidant defense system and the immune system [41,42,43]. Selenoproteins have anti-cancer activity through antioxidant effects and DNA stabilization (deoxyribonucleic acid) [44]. In studies on cell lines, selenium demonstrates apoptotic and antiproliferation activity in malignant and healthy cells [45]. On the other hand, a high dose of selenium compounds inhibits neoplastic growth by production of reactive oxygen species [46]. Treatment with a high amount of sodium selenite selectively eliminates cancer cells by generating free radicals [47]. The results from a phase 1 study indicated it was safe and efficient to treat patients with metastatic cancer with selenium in combination with palliative chemotherapy [48,49].

To our knowledge, there are no data which assess the relationship between blood selenium level and the effectiveness of NAC.The aim of the current analysis is to evaluate the impact of blood selenium levels on achieving pCR in HER2-positive andtriple-negative breast cancer patients treated with NAC.

## 2. Materials and Methods

### 2.1. Study Population

**Figure 1:**
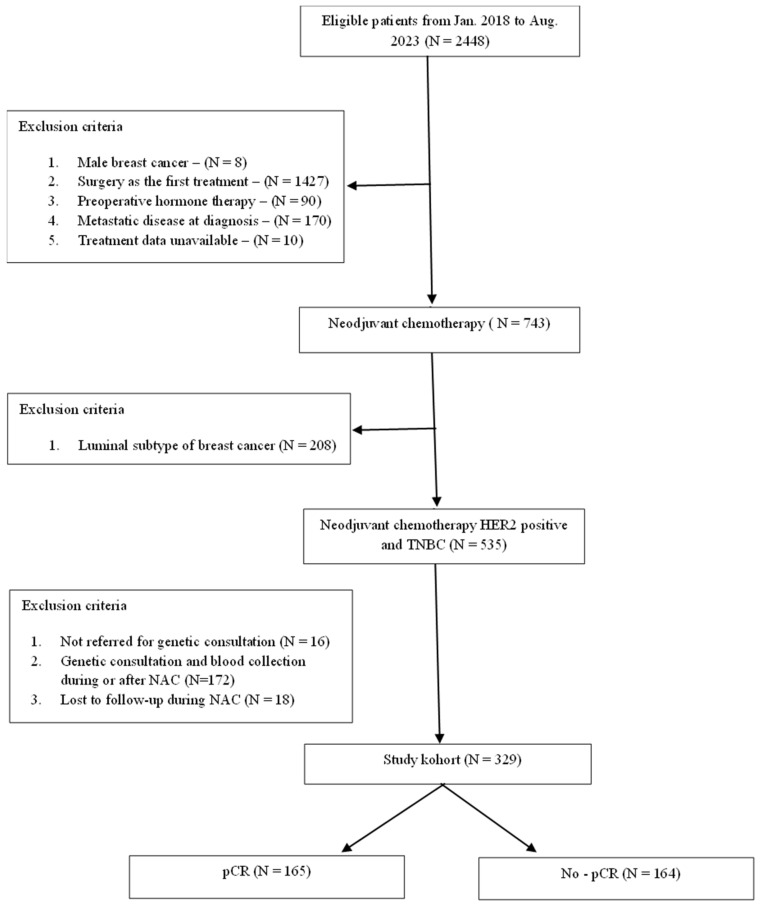
Study case selection procedure.

This was a non-interventional, prospective study. The study population consisted of women with breast cancer diagnosed between January 2018 and August 2023 who were treated with NACat either of two hospitals in Opole and Zielona Góra, Poland. Patients were selected according to the following criteria: 18 years old or older at time of diagnosis; female sex; diagnosis of invasive breast carcinoma; stage cT1-4N0-3M0; receipt of NAC followed by surgery (according to TNM v.VII) [50]; and TNBC or HER2-positive subtypes. Patients were excluded if they had simultaneous bilateral breast cancer or metastatic disease at diagnosis (stage IV cancer). Figure 1 displays the case selection procedure.

A blood sample was collected and stored after the patients signed written consent. Each patient was tested for Polish founder mutations in *BRCA1* (c.5263_5264insC; c.4035delA; c.181T>G, c.3700_3704delGTAAA, c.68_69delAG), *CHEK2* (c.100delC, c.444+1G>A, del5395(ex10-11del), c.470C>T), and *PALB2* (c.509_510delGA, c.172_175deITTGT) genes.

Clinical data were obtained from patient interview and from medical records. The clinicopathological features included tumor size, clinical stage, histological type, tumor grade, estrogen receptor (ER), progesterone receptor (PR), HER2 status, Ki67 status, age of diagnosis, body mass index (BMI), menopausal status, and type of chemotherapy regimen.

HER2-positive patients received anti-HER2 treatment based on trastuzumab or trastuzumab and pertuzamab. Patients with triple-negative breast cancer (TNBC) were treated with a range of combination chemotherapies. In the analysis, 329 patients were included. The patients were classified in two phenotypic subgroups: triple-negative (TNBC) and HER2-positive. HER2 status was determined through HercepTest. HER2 status was considered negative if equal to 0 or 1+ by immunohistochemistry (IHC) or if 2+ by IHC and not amplified (FISH/SISH/CISH). HER2 status was considered positive if 3+ by IHC or 2+ by ICH and amplified (FISH/SISH/CISH) [51]. Among those with HER2-positive breast cancer, positive ER or PR was defined as the presence of at least 1% positive cancer cells among the total number cells examined by IHC analysis [52]. pCR was defined as no invasive cancer cells in primary lesion and lymph nodes at surgery after NAC [9].

The study was conducted in accordance with the Declaration of Helsinki and was approved by the Ethics Committee of the Pomeranian Medical University in Szczecin.

### 2.2. Analytical Procedures

We collected a blood sample from each participating patient. Patients were asked to fast for at least four hours prior to giving blood. The blood samples were obtained from fasting individuals through venipuncture using the Vacutainer^®^ System (BD#368381, Plymouth, UK). Blood was carefully divided into new cryovials and then frozen at −80 °C until analysis.

The elemental composition of the samples was analyzed usinginductively coupled plasma mass spectrometry (ICP-MS) with the NexION 350D instrument (PerkinElmer, Norfolk, VA, USA). Selenium isotope ^78^Se was selected forquantification. The KED (Kinetic Energy Discrimination) mode was applied for element determination, and rhodium served as an internal standard to accountfor instrument drift and matrix effects. Further detailsregarding the specific parameters of the NexION 350D instrument used in the measurements are availableupon request. During analysis, the blood samples were diluted 40-fold with blank reagent (70 µL blood/2730 µL buffer).

The blank reagent consisted of high-purity water (>18 MΩ), TMAH (AlfaAesar, Kandel, Germany), Triton X-100 (PerkinElmer, Shelton, CT, USA), and ethyl alcohol (Merck, Darmstadt, Germany).

The calibration curve standards were prepared by diluting the stock solution of 10 mg/L Multi-element Calibration Standard 3 (PerkinElmer Pure Plus, Shelton, CT, USA) with the blank reagent. The calibration method was matrix-matchedwith the correlation coefficients for the calibration curve, consistently exceeding 0.999.

The accuracy and precision of the measurements were assessed using certified reference materials (CRMs): ClinChek^®^ Plasmonorm Whole Blood Level 1 (Recipe, Munich, Germany) and Seronorm Whole Blood Level 2 (Sero, Hvalstad, Norway). Technical information, plasma operating settings, and mass spectrometer acquisition parameters are availableupon request. The testing laboratory participates in the independent external quality assessment scheme, QMEQAS (Quebec Multielement External Quality Assessment Scheme), organized by the Institut National de SantéPublique du Québec.

### 2.3. Statistical Analysis

We assigned patients to one of three groups of equal size based on the blood selenium levels. We considered the subgroup of patients with the lowest selenium levelsas the reference group. We estimated the pCR rate for various subgroups of women based on clinical and pathological characteristics. We performed logistic regression analysis to identify variables that were significant predictors of pCR. In the multivariate analysis, we included variables with a *p*-value ≤ 0.1 in the univariate analysis and we considered a *p*-value ≤ 0.05 to be statistically significant. The differences between selenium levels according to clinical factors and type of chemotherapy were assessed for statistical significance using Student’s test and one-way ANOVA. The analysis was conducted using TIBCO Software Inc. (2017) (Palo Alto, CA, USA) and Statistica (data analysis software system), version 13 (StatSoft, Krakow, Poland; http://statistica.io, accessed on 10 November 2020).

## 3. Results

### General Characteristics

There were 329 breast cancer patients included in this study. The median age of diagnosis was 54.8 years (range 25–85 years), 58.4% of patients were postmenopausal, 31.9% were overweight and 26.2% were obese. Germline mutations were detected in 48 patients (14.6%): *BRCA1* in 22 patients (6.7%) a *CHEK2* in 18 patients (5.5%) and *PALB2* in 8 patients (2.4%). In total, 172 of the 329 patients had positive lymph nodes by clinical examination (52.3%) and 99 patients had a tumor size above 5.0 cm, by imaging (30.1%). Overall, 146 (44.4%) of the patients had TNBC and 183 (55.6%) of patients had HER2-positive tumors. Of the HER2-positive patients, 119 (65.4%) were ER-positive. Characteristics of the patients and mean selenium levels are presented in Table 1.

The probability of achieving a pCR according to clinical subtype is presented in Table 2. It is of note that the probability of achieving a pCRwas significantly different according to tumor size, Ki67 status, BMI, and selenium level.

The pCR rate was 39.0% for those in the lowest tertile of selenium, 52.0% for those in the middle tertile, and 59.0% for those in the highest tertile. For the entire study group, the adjusted hazard ratio for achieving a pCR was 2.75 (95% OR 1.53–4.95, *p* = 0.001) for those in the highest tertile of selenium compared to those in the baseline (reference) tertile. For those with HER2-positive cancer, the adjusted hazard ratio for achieving a pCR was 1.65 (95% OR 0.70–3.88, *p* = 0.25) for those in the highest tertile of selenium compared to those in the baseline (reference) tertile. For those with TNBC, the adjusted hazard ratio for achieving a pCR was 6.70 (95% OR 2.18–20.56, *p* = 0.001) for those in the highest tertile of selenium compared to those in the baseline (reference) tertile.

We also compared an additional analysis of the pCR rate between those in the lowest selenium subgroup and the combined intermediate and highest selenium group, and the difference between the groups was also statistically significant (39.0% vs. 56.0%; *p* = 0.004).

The mean selenium level for patients who achieved a pCR was 104.0 μg/L and for those who did not achieve a pCR was 99.7 μg/L; *p* = 0.01, (Table 1). Predictors of pCR are presented in Table 2.

The difference in response rates for the different levels of selenium was not due to differences in the chemotherapy regimen given (Table 3).

## 4. Discussion

In this study of breast cancer patientsundergoing neoadjuvant chemotherapy, we observed a significantly higher pCR rate in the group of patients with the highest selenium level compared to the group with the lowest level (OR = 2.75; *p* = 0.001). In the group with the highest selenium level, the pCR rate was 59.0%, and in the subgroup with the lowest level, it was 39.0% (*p* = 0.003). The association was much stronger for those with triple-negative cancer (OR = 6.70 (95% OR 2.18–20.56, *p* = 0.001)) than for those with HER2-positive cancer (OR = 1.65 (95% OR 0.70–3.88, *p* = 0.25)).

To our knowledge, this is the first study to demonstrate an association between selenium levels in whole blood and pCR after neoadjuvant chemotherapy. We have previously reported that low serum selenium levels are associated with a decrease in 5-year and 10-year survival in breast cancer patients [39,40]. In the previous study, we determined the level of selenium in serum, and in the current study, we determined it in whole blood. Determining selenium in whole blood is technically simpler, does not require blood centrifugation and plasma separation, and requiresless time from staff.

In the study, we selected patients with HER2-positive and TNBC breast cancer as the study group because in these subtypes of breast cancer, achieving pCR after NAC strongly correlates with prognosis and survival [9,53].

Our study is a non-interventional study of real-world data (RWD). Patients received many different treatment regimens. In the subset of HER2-positive patients, all patients receivedHER2-targeted therapy, and 64.3% of patients received dual blockade with pertuzamab and trastuzumab. Most patients (86.3%) received treatment with anthracyclines and taxanes (paclitaxel or docetaxel). NAC without anthracyclines (TCH; TCHP) was received by 13.7% of patients. The rate of pCR achieved in this subgroup was 54.1%. In the phase II clinical trial TRAIN-2, using double anti-HER2 blockade, a higher pCR rate of 67% was found in the subgroup with anthracyclines and 68% in the subgroup without anthracyclines [54]. A similar pCR rate (52.7%) was demonstrated in a study of 355 patients with HER2-positive breast cancer treated with dual HER2 blockade as part of daily clinical practice [55].

In our study, in the multivariate analysis, the following factors were statistically significantly associated with achieving pCR: primary tumor size, BMI, Ki67 level, and selenium level in whole blood. We did not observe an association between the presence of lymph node metastases and the probability of achieving pCR. These data are consistent with others [50,55,56,57].

Another important factor was the Ki67 value. In this study, we divided patients into three subgroups with low, intermediate, and high Ki 67 values. We considered 30% as a low value and above 50% as a high value. In the literature, the relationship between the Ki67 level and the pCR rate was observed, but in these studies, different Ki67 values were considered as the cut-off point (14%, 20%, 30%); therefore, it is difficult to compare the data obtained in these studies [50,55,56,57,58].

In our study, patients with a normal BMI were more likely to achieve pCR compared to obese patients (HR = 2.42; *p* = 0.007). Single studies published so far have described the unfavorable impact of obesity and overweight on achieving pCR, especially in estrogen receptor-positive breast cancer patients [35,59].

We did not find any differences in the average selenium level between subgroups of patients with factors that could have influenced the achievement of pCR (primary tumor size, axillary lymph node metastases, Ki67 value).

This indicates that the selenium level is an independent factor influencing pCR after NAC; accordingly, the odds ratios for pCR according to selenium level were similar in the univariate and multivariate analyses.

We are unsure of the mechanism that causes selenium to have an influence on the pCR rate after NAC. Selenium is an essential component of several major metabolic pathways, including the antioxidant defense system and the immune system, and selenium is incorporated into 30 different selenoproteins [41,42,43]. Selenium influences both the innate and adaptive immune systems [60]. A relationship with selenium supplementation and the protection of neutrophils against endogenous oxidative stress has been described [61]. Serum selenium concentrations were positively associated with the increased number and activity of natural killer (NK) cells [62]. Selenium intake increases M1 macrophages with antitumor activity in the tumor microenvironment [63]. In addition, selenium also increases the proliferation and lytic activity of CD8+ T-lymphocytes against tumor cells [64]. On the other hand, treatment with a high amount of sodium selenite selectively eliminates cancer cells by generating more free radicals than that of normal cells without systemic Se toxicity [47]. The results from a phase 1 study indicated that selenium was safe and efficient for treating patients with metastatic cancer in combination with palliative chemotherapy [48,49]. Additionally, a clinical study of patients with cervical cancer treated with chemoradiotherapy showed that selenium supplementation reduced the frequency of hematological side effects [65].

Several published studies have assessed the effect of selenium components on breast cancer cells. In a preclinical study, methylselenic acid was shown to enhance the efficacy of paclitaxel in the treatment of triple-negative breast cancer. The synergism was attributable to more pronounced induction of caspase-mediated apoptosis, arrest of cell cycle progression at the G2/M checkpoint, and inhibition of cell proliferation [66]. In another study, it was shown that di and triselenoesters are effective therapeutic agents for inhibiting multidrug resistance proteins in breast cancer cells, which may increase the sensitivity of these cells to chemotherapy [67]. In another study, Omega-3 fatty acids from fish oil and selenium with doxorubicin compared with doxorubicin alone resulted in lower tumor sizes and reduced overall metastasis rates, lower GPR-40 mRNA levels, and higher expression of all selenoproteins. Doxorubicin-FO/Se combination treatment decreased expression of membrane EGFR and FGFR, down-regulated downstream PI3K/AKT/mTOR, MAPK/ERK, and JAK2/c-Src/STAT3 signaling, increased tumor suppressor PTEN/TSC1/TSC2 expression and P53 activation, and suppressed oncogenic transcription factor expression [68]. In turn, in the case of HER2-positive breast cancer, selenium inhibits growth of trastuzumab-resistant human breast cancer cells via downregulation of Akt and beclin-1 [69]. The combination of trastuzumab with selenium components has also been shown to be active against cells resistant to trastuzumab alone in an in vitro study on HER2-positive breast cancer cell lines [70].

There are several limitations of our study. Our study is a non-randomized observational study. This concerns real-world data; therefore, decisions on dose reduction, treatment interval, and type of chemotherapy used were made by the NAC physicians and were not based on strictly defined protocols. For this reason, patients received different types of chemotherapy under NAC. We included only patients with HER2-positive and TNBC subtypes of breast cancer and performed a joint analysis of both subtypes, but the sample sizes were small for the subgroups. There were too few patients with predisposing mutations to assess these groups individually. We did not have sufficient follow up time to consider breast cancer-specific survival.

Finally, our results suggest there may be a potential benefit for selenium supplementation in breast cancer patients with low selenium levels for whom NAC is planned. So far, selenium supplementation has been tested in a small study in a group of patients with cervical cancer undergoing chemoradiotherapy. In that study, selenium supplementation reduced the incidence of hematological side effects [65]. There are currently no clinical trials on selenium supplementation in patients with breast cancer receiving NAC. Testing this hypothesis in breast cancer patients requires planning interventional studies with random selection in subgroups. Due to the observational nature of our study, the data obtained should be confirmed in a prospective observation.

In the future, we plan to enlarge the study group and conduct PFS and OS analysis, but this requires extending the observation period. Another planned analysis is the assessment dose intensity of chemotherapy and chemotherapy-related toxicities depending on the selenium level in the blood.

## 5. Conclusions

We conclude that HER2-positive and TNBC breast cancer patients with a selenium blood level above 107.19 μg/L before starting NAC have a higher pCR rate than patients with selenium levels below 94.29 μg/L. The association is particularly strong for the triple-negative subtype.

## Figures and Tables

**Table 1:** Study population characteristics.

Clinicopathological Parameters		*n* = 329	%	Mean Selenium Level ± SD	*p*-Value
Age mean (range)	54.8 (25–85)				
	≤50	126	38.3	100.4 ± 13.9	0.18
	>50	203	61.7	102.8 ± 17.0	
BRCA1 status					
	Positive	22	6.7	96.3 ± 22.4	0.09
	Negative	307	93.3	102.3 ± 15.3	
CHEK2 status					
	Positive	18	5.5	100.3 ± 15.0	0.67
	Negative	311	94.5	101.9 ± 16.0	
PALB2 status					
	Positive	8	2.4	95.5 ± 15.8	0.25
	Negative	321	97.6	102.0 ± 15.9	
Menopausal status					
	Premenopusal	137	41.6	100.2 ± 13.6	0.11
	Postmenopauzal	192	58.4	103.0 ± 17.3	
Smoking status					
	Current	72	21.9	99.7 ± 15.2	0.18
	Past	57	17.3	104.8 ± 17.9	
	Never	176	53.5	102.8 ± 15.7	
	Missing	24	7.3	95.6 ± 11.7	
BMI					
	≤24.9	138	41.9	100.5 ± 15.4	0.40
	25.0–29.9	105	31.9	103.3 ± 16.2	
	≥30	86	26.2	102.3 ±16.5	
Tumor size					
	≤20 mm	28	8.5	102.3 ± 19.1	0.90
	21–50 mm	202	61.4	101.5 ± 15.4	
	≥51 mm	99	30.1	102.4 ± 16.2	
Clinical node status					
	Positive	172	52.3	100.8 ± 14.9	0.22
	Negative	157	47.7	103.0 ± 16.9	
Histology					
	Ductal, NST	320	97.3	101.8 ± 15.8	0.61
	Other	9	2.7	104.5 ± 20.3	
Tumor grade					
	G1	4	1.2	99.4 ± 9.7	0.74
	G2	150	45.6	102.4 ± 15.2	
	G3	169	51.4	101.1 ± 16.6	
	Missing	6	1.8	112.6 ± 17.8	
ER status					
	Negative	210	63.8	102.8 ± 16.4	0.15
	Positive	119	36.2	100.2 ± 15.0	
PR status					
	Negative	249	75.7	102.3 ± 15.9	0.37
	Positive	80	24.3	100.5 ± 15.9	
HER2 status					
	Positive	183	55.6	101.8 ± 14.7	0.92
	Negative	146	44.4	102.0 ± 17.3	
Ki 67 status					
	0–30%	68	20.7	102.4 ± 17.8	0.96
	31–50%	73	22.2	101.9 ± 13.9	
	51–100%	185	56.2	101.8 ± 16.1	
	Missing	3	0.9	94.0 ± 1.6	
pCR status					
	pCR	165	50.2	104.0 ± 16.0	0.01
	no pCR	164	49.8	99.7 ± 15.6	

**Table 2:** Predictors of pCR: Univariable and multivariable analysis.

Clinicopathological Parameters		*n* = 329	% PCR	Univariable Odds Ratio		*p*-Value	Multivariable Odds Ratio		*p*-Value
				OR	(95% OR)		OR	(95% OR)	
Age (years)									
	≤40	52	65.4	2.01	(1.03–3.93)	0.04	1.39	(0.65–2.96)	0.40
	41–50	74	48.6	1.01	(0.57–1.79)	0.97	0.87	(0.46–1.67)	0.68
	51–60	77	44.2	0.84	(0.48–1.49)	0.56	0.67	(0.36–1.27)	0.22
	≥61	126	48.4	Reference			Reference		
Tumor size									
	≤20 mm	28	71.4	3.26	(1.31–8.10)	0.01	3.03	(1.15–7.99)	0.02
	21–50 mm	202	50.5	1.33	(0.82–2.16)	0.25	1.61	(0.94–2.74)	0.08
	≥51 mm	99	43.4	Reference			Reference		
Breast cancer subtype									
	HER2-positive	183	54.1	Reference					
	TNBC	146	45.2	0.70	(0.45–1.08)	0.11			
ER status									
	Positive	119	46.2	Reference					
	Negative	210	52.4	1.28	(0.82–2.00)	0.28			
PR status									
	Positive	80	41.3	Reference			Reference		
	Negative	249	53.0	1.61	(0.97–2.68)	0.07	1.67	(0.95–2.96)	0.08
Ki67 status									
	0–30%	68	32.4	Reference			Reference		
	31–50%	73	53.4	2.40	(1.21–4.76)	0.012	2.18	(1.04–4.55)	0.04
	51–100%	185	55.1	2.57	(1.43–4.61)	0.002	2.50	(1.33–4.69)	0.004
	missing	3	66.7						
Grade									
	1	4	25.0	Reference					
	2	150	48.7	2.84	(0.29–27.97)	0.37			
	3	169	51.5	3.18	(0.33–31.22)	0.32			
	missing	6	66.7						
Mutation status									
	None	281	48.8	Reference					
	BRCA1	22	59.1	1.52	(0.63–3.67)	0.35			
	CHEK2	18	55.6	1.31	(0.50–3.43)	0.58			
	PALB2	8	62.5	1.75	(0.41–7.47)	0.45			
Lymph nodes									
	Negative	157	49.0	0.92	(0.60–1.42)	0.70			
	Positive	172	51.2	Reference					
BMI									
	<24.9	138	59.4	2.35	(1.36–4.08)	0.002	2.39	(1.27–4.49)	0.007
	25.0–29.9	105	47.6	1.46	(0.82–2.61)	0.20	1.57	(0.84–2.94)	0.16
	≥30	86	38.4	Reference			Reference		
Selenium level *									
	Tertile 1	110	39.0	Reference			Reference		
	Tertile 2	109	52.3	1.71	(1.00–2.92)	0.05	1.72	(0.97–3.06)	0.07
	Tertile 3	110	59.0	2.25	(1.31–3.86)	0.003	2.75	(1.53–4.95)	0.001

* Selenium Tertile 1 range 62.9–94.2 µg/L; Selenium Tertile 2 range 94.3–107.1 µg/L; Selenium Tertile 3 range 107.2–167.8 µg/L.

**Table 3:** Mean selenium level according to NAC regimen given.

NAC Regimens	*n* = 329	%	Mean Selenium Level ± SD	*p*
				0.65
AC-Paclitaxel	18	5.5	97.17 ± 15.02	
AC-Paclitaxel + Carboplatin	13	4.0	100.78 ± 24.00	
AC(dd)-Paclitaxel	55	16.7	102.64 ± 20.61	
AC(dd)-Paclitaxel + Carboplatin	48	14.6	104.58 ± 11.10	
AC/EC-Docetaxel/Paclitaxel + Herceptin	53	16.1	100.83 ± 13.76	
AC/EC Docetaxel + Herceptin + Pertuzumab	69	21.0	103.053±14.34	
AC(dd)-Paclitaxel+Herceptin	2	0.6	92.49±20.56	
AC(dd)-Docetaxel/Paclitaxel+Herceptin+Pertuzumab	31	9.4	102.55±15.16	
Docetaxel+Caroplatin+Herceptin	9	2.7	99.95±16.42	
Docetaxel+Caroplatin+Herceptin+Pertuzumab	16	4.9	103.38±16.92	
Other	15	4.6	95.18 ± 16.38	

Notes: AC = doxorubicin + cyclophosphamide; AC(dd) = doxorubicin plus cyclophosphamide administered every 14 days; EC = epirubicin + cyclophosphamide.

## Data Availability

The data presented in this study are available on request from the corresponding author as the source data are materials for subsequent publications in the habilitation process.

## Abbreviations

AKT: set of three serine/threonine kinase; BRCA1: breast cancer 1 gene; BMI: body mass index; CHEK2: checkpoint kinase 2 gene; c-Src: C-terminal Src kinase; DFS: disease-free survival; EGFR: epidermal growth factor receptor; ER: estrogen receptor; ERK: extracellular signal-regulated kinase; FGFR: fibroblast growth factor receptor; HER2: human epidermal growth factor receptor 2; JAK2: Janus kinase 2; MAPK: mitogen-activated protein kinase; mTOR: mammalian target of rapamycin kinase; NAC: neoadjuvant chemotherapy; OR: odds ratio; PALB2: partner and localizer of *BRCA2* gene; pCR: complete pathological response; p53:tumor protein P53; PR: progesterone receptor; PTEN: phosphatase and tensin homolog; RWD: real-world data; Se: selenium; STAT3: signal transducer and activator of transcription 3; TSC1: Tuberous sclerosis 1 kinase; TSC2: Tuberous sclerosis 2 kinase.
